# Evidence-Based mHealth Chronic Disease Mobile App Intervention Design: Development of a Framework

**DOI:** 10.2196/resprot.4838

**Published:** 2016-02-16

**Authors:** Calvin C Wilhide III, Malinda M Peeples, Robin C Anthony Kouyaté

**Affiliations:** ^1^ WellDoc Inc Baltimore, MD United States

**Keywords:** mHealth, mobile applications, mobile app design, chronic disease, diabetes, mHealth framework, behavioral intervention, intervention design, mHealth implementation, telemedicine

## Abstract

**Background:**

Mobile technology offers new capabilities that can help to drive important aspects of chronic disease management at both an individual and population level, including the ability to deliver real-time interventions that can be connected to a health care team. A framework that supports both development and evaluation is needed to understand the aspects of mHealth that work for specific diseases, populations, and in the achievement of specific outcomes in real-world settings. This framework should incorporate design structure and process, which are important to translate clinical and behavioral evidence, user interface, experience design and technical capabilities into scalable, replicable, and evidence-based mobile health (mHealth) solutions to drive outcomes.

**Objective:**

The purpose of this paper is to discuss the identification and development of an app intervention design framework, and its subsequent refinement through development of various types of mHealth apps for chronic disease.

**Methods:**

The process of developing the framework was conducted between June 2012 and June 2014. Informed by clinical guidelines, standards of care, clinical practice recommendations, evidence-based research, best practices, and translated by subject matter experts, a framework for mobile app design was developed and the refinement of the framework across seven chronic disease states and three different product types is described.

**Results:**

The result was the development of the Chronic Disease mHealth App Intervention Design Framework. This framework allowed for the integration of clinical and behavioral evidence for intervention and feature design. The application to different diseases and implementation models guided the design of mHealth solutions for varying levels of chronic disease management.

**Conclusions:**

The framework and its design elements enable replicable product development for mHealth apps and may provide a foundation for the digital health industry to systematically expand mobile health interventions and validate their effectiveness across multiple implementation settings and chronic diseases.

## Introduction

The promise of mobile technology to revolutionize health care services and patient self-management behavior for chronic disease has intrigued practitioners and researchers for well over a decade [1]. The attributes that give it an advantage over other information and communication technologies are its popularity, mobility, and technological capabilities [2]. Mobile health (mHealth) interventions can benefit health care by reaching people in resource-poor settings, delivering interventions to large numbers of people, claiming people’s attention when it is most relevant, enabling access to and delivery of customized support, and providing low cost interventions [3]. Mobile technology offers new capabilities that can help to drive important aspects of chronic disease management at both an individual and population level, including the ability to deliver real-time interventions that can be connected to a health care team.

Researchers, technology companies, health care companies, health plans, and pharma have been exploring these capabilities across different chronic disease management programs and models**.** In order to design mHealth solutions, practitioners and researchers can draw from innovation, best practices, theory, and evidence. When building different mHealth products for a variety of settings and programs, the type of mHealth product developed is dictated by the objectives deemed necessary for the population being served. For example, building mHealth solutions that provide comprehensive management of chronic disease will differ from building those that support one component of chronic disease management in an existing health care program.

Over the past decade, the evidence base indicating the efficacy and effectiveness of these mHealth technology solutions has been growing for the management of chronic disease. However, evidence of their effectiveness has been inconclusive [3-5]. Some literature has provided preliminary evidence regarding the utility of linking mHealth into existing health care models to help drive improved outcomes [6-11]. mHealth apps have utilized many intervention strategies such as tracking and texting to offer more comprehensive management support [8,12]. The inconclusive findings highlight the importance of the following questions: which aspects of mHealth work for which diseases, and for whom, to achieve which outcomes?

In order to answer these questions, a systematic approach to designing mHealth apps to support health programs and services becomes vitally important. To date, there is very little to guide such a process [13], which facilitates translation of the emerging mHealth science and literature into scalable, replicable, evidence-based mHealth solutions that can be adapted to multiple, real-world health care settings and systematically evaluated. Through the experience of developing mHealth products for different health care settings, there was a unique opportunity to develop and test systematic approaches for designing and developing mHealth interventions. Informed by the Chronic Care Model [14], health behavior models and theories, clinical and behavioral program best practices [15-18], and health care outcomes, an app design framework evolved. The purpose of this paper is to discuss the identification and development of an app intervention design framework, and its subsequent refinement through development of various types of mHealth apps for chronic disease.

## Methods

### Phase 1: Developing the Initial Framework (June to August 2012)

As a first step in creating a systematic process for app intervention design, the Clinical Programs and Research subject matter experts (SME) identified the strategic, intervention, and program domains that were the foundation of a telephonic disease management program that had demonstrated positive outcomes [19]. In that program, patient interventions were delivered telephonically by a case manager. For mobile app intervention development, we needed to understand how to leverage the anywhere, anytime, contextual capabilities of mobile technology, as well as employ existing evidence and expertise to modify, adapt, and incorporate traditional interventions in the context of mobile app product design. In collaboration with the Behavior Change SME and other clinicians, the initial framework (strategic, intervention, and program domains) was translated into a framework that was specific to mobile app intervention design.

The strategic domains were expanded to include value drivers, outcomes and metrics, and program objectives as defined by key stakeholders. Since mobile health intervention development is still in an early stage, it was essential to understand what types of intervention(s) could be delivered through an app and how success would be measured. For example, tracking a metabolic measure such as blood pressure or weight required different features than providing tailored behavioral support that could impact clinical and behavioral outcomes.

Through the collaborative process, the intervention domain was expanded to integrate appropriate clinical and behavioral components that were informed by clinical guidelines, standards of care [20], clinical practice recommendations, evidence-based research, best practices, and programmatic expertise for chronic disease. The clinical, program, and behavioral experts reviewed all the clinical resources and leveraged existing programmatic guidelines [15-18,21] and experience for developing behavior change strategies. Three intervention domains were added: essential behaviors (supporting actions and determinants), multidimensional profiles, and evidence-based clinical and behavioral interventions. Together these domains make up the intervention plan. This plan then informs the design of the specific product features and content that support the intervention(s) and drive outcome(s).

### Phase 2: Applying and Refining the Framework (September 2012 to October 2013)

After the initial development, the framework was applied to design three types of mHealth products (apps) providing a range of chronic disease support and management: (1) direct-to-consumer apps providing targeted intervention(s) such as tracking, reminders, and data display (eg, symptom tracker); (2) program apps developed for “single-focus programs” (eg, low back pain); and (3) prescription apps providing comprehensive chronic disease support through numerous features and capabilities linking the patient and health care team (eg, mobile prescription therapy). Initially, the framework was applied to type 2 diabetes and refined. With each application of the framework to a new disease state and product/program, domains were validated and components for each domain were refined, such that an initial taxonomy of app intervention design evolved.

### Phase 3: Finalizing the Framework (March 2013 to June 2014)

Through collaboration with clinical informatics SMEs and software developers, and the application of the framework domains to the development of different types of mobile apps, the finalized framework and taxonomy for an mHealth app intervention design evolved. The taxonomy reflected the framework domains and associated design elements for each domain attribute.

## Results

### The Design Framework

**Figure 1:**
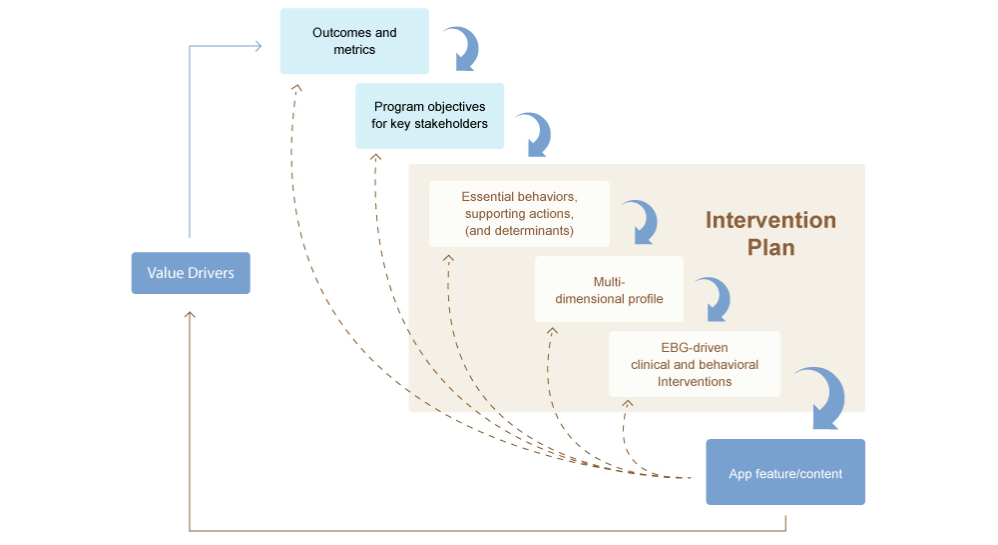
mHealth waterfall process.

The Chronic Disease mHealth App Intervention Design Framework resulted from an iterative process in which the framework was applied, evaluated, and refined during the design and development of different types of apps for 7 chronic diseases. The framework includes 7 domains to guide the development of apps: 3 strategic, 3 intervention design, and 1 product feature(s) and content. As the work for each domain is completed, the output is fed into the next domain in what is described as a “waterfall process” (Figure 1). This process guides both the development of the product features (downward arrows) and the evaluation (dotted upward arrows) of their effectiveness in driving desired outcomes. Application of the framework in a systematic way results in the identification of key features and content to be included in mHealth products. The specific domains evolved because they provide a coherent means of explicitly describing the logic behind major decisions related to the features and content included in mHealth products. They also guide the measurement of the process, outcomes, and impact that the mHealth app brings to the product/program. Application of the framework results in apps that are either direct-to-consumer tracking apps, “single-program” apps, or mobile medical apps regulated by the Federal Drug Administration (FDA). Insights gained in relation to design decisions for different types of apps demonstrated the value of the framework. A comparative analysis of different apps will be presented in subsequent publications.

### The Framework

The following is a description of the framework and its 7 domains. For each domain, we present a definition and how the domain was derived. The first 3 domains (value drivers, outcomes, and program objectives) are strategic and were determined through interaction with external stakeholders.

#### Value Drivers

##### Definition

Value drivers are the entities that increase the value of a product or service [22]. Examples for an mHealth product can include improved health for an individual or population, improved access to care, or reduced health care costs.

##### How Derived

Value drivers were initially determined by analyzing gaps and challenges in chronic disease management at the population level, in service delivery, and in patient self-management. For each mHealth product, value drivers were informed by the overall goals and objectives of the program and by the unique capabilities that an mHealth app could contribute to the improvement of chronic disease management, service delivery, and/or patient self-management. It was also necessary to take into account the setting in which the mHealth solution would be deployed (eg, primary health care, large employer-based insurance program, large pharmaceutical). Value drivers set the course for the development of the mHealth solution, and in turn, dictated subsequent elements of the “waterfall.”

#### Outcomes and Metrics

##### Definitions

Outcomes are the desired results of the program, and generally can be short (eg, knowledge, attitudes), intermediate (eg, self-care behaviors), or long term (eg, A1c) depending on the objective, length of the program, and expectations of the program or intervention(s) [23]. Metrics are a means of measurement and are aligned to industry-recognized quality metrics to facilitate a program or providers’ ability to demonstrate that intervention(s) have an impact.

##### Derivation

Guided by the designated value drivers for each program, outcomes and metrics were aligned with those of the chronic disease program being supported. National guidelines, standards of care, published literature, and meta-analyses were common sources for identifying standard outcomes and metrics for each product.

#### Program Goals for Key Stakeholders

##### Definition

Key stakeholders are those users that the system is designed to support directly. The program goals for key stakeholders represent what the intended users should accomplish via product use. Typically, they signify fundamental, long-range achievements based on engagement with the mHealth product and are broad general statements [24].

##### Derivation

Based on the value drivers, outcomes, and metrics, key stakeholders and corresponding program goals for those stakeholders were identified by interdisciplinary teams responsible for developing the mHealth programs and products.

#### Intervention Plan

The intervention domain was expanded to include 3 subdomains that resulted from the translation and application of current behavioral and clinical evidence, as well as subject matter expertise to the real-time, contextual capabilities of mobile technology: essential self-management behaviors, multidimensional profiles, and integrated clinical/behavioral interventions. These 3 domains were grouped together as the intervention plan.

These domains guided the design of integrated clinical and behavioral interventions that target behaviors known to improve clinical outcomes. Behavioral interventions were designed based on clinical contexts to guide intended value drivers and outcomes that uniquely address the chronic disease of interest. Several of the most commonly used behavior change theories guided/informed these domains [25]; specifically, determinants addressed through product design included the social ecologic model [26], the health belief model [27], social cognitive theory/social learning theory [28], theory of reasoned action and planned behavior [29], and the transtheoretical stages of change model [30]. Disease specific guidelines, professional best practices, and associated behavioral research informed the evidence that was translated into the intervention design. Systematic application of these domains resulted in an intervention plan that was used to guide decisions about product features and content.

#### Behavioral Domains, Essential Behaviors, Supporting Actions and Determinants

##### Definition

Behavioral domains are the categories of self-management behaviors, and encompass essential behaviors, their associated supporting actions, and determinants. An essential behavior is a behavior “...that should be emphasized through program interventions because of its impact on public health, its measurability, and its feasibility to be performed by patients, caretakers and/or health workers” [17]. Ideally, research has demonstrated that the behavior is associated with improvements in clinical outcomes or longer-term health impact or quality of life. Supporting actions, or sub-behaviors, are the combined small, do-able actions that comprise the essential behaviors [31]. Determinants identified are predictors of behavior change or of present behavior [32].

##### Derivation

Based on the program objectives for key stakeholders, standards and guidelines were used to inform the identification of behavioral domains supporting selected clinical outcomes (eg, American Association of Diabetes Educators 7 behavioral domains; AADE7) [21]. When available, published research demonstrating correlations of behaviors with clinical outcomes [19] was utilized to identify essential behaviors. Interdisciplinary teams of clinical, behavioral, and user interface/user experience (UI/UX) SMEs identified a broad set of supporting actions for each essential behavior and noted specific actions that could be supported through mHealth interventions. Finally, theory, published literature, formative research, and marketing research provided insights on behavioral determinants.

#### Multidimensional Profile

##### Definition

The multidimensional profile is a segmentation approach. It drives the individualization of a user’s experience through the customized delivery of interventions via features and tailored message content. Customized delivery can be achieved via preferences set by users or automated, dynamic, and adaptive delivery of interventions based on analysis of longitudinal patterns of data [8]. Content tailoring includes targeted messages based on demographic characteristics, personalization (eg, incorporating first name into messages), or tailoring content in response to assessments, captured data, or the context in which data was captured (eg, bedtime blood glucose [BG] reading) [33].

##### Derivation

Once goals and essential behaviors were determined, formative market research and the published literature informed the various dimensions of a user profile that were relevant to the essential behaviors. Dimensions included but were not limited to clinical, behavioral, psychosocial, contextual, and personal factors.

#### Evidence-Based Clinical/Behavioral Interventions

##### Definition

Evidence-based interventions are those that have been identified as effective for achieving outcomes, and are best practices, which have been peer-reviewed and evaluated for effectiveness in improving health outcomes [34].

##### Derivation

These interventions were based on program goals for stakeholders, essential behaviors, and user profiles, and whenever available, the most rigorous quality of evidence was utilized [34]. Interventions were first identified through an analysis of clinical guidelines, standards of care, evidence-based public health programs, medicine, and health care, as well as meta-analyses and systematic reviews of intervention research. However, given the emerging nature of mobile technology, other types of evidence were also considered, including state-of-the-art practices, and best and emerging practices for innovations [34]. Interventions were categorized based on their strategic intent (eg, monitoring support or education support) for incorporation into the framework [35].

#### App Feature/Content

Following the “waterfall” process, intervention plans were then translated into product features and content identified to deliver optimal outcomes to meet product/program objectives.

##### Definition

Product features, functionality, and content are designed to deliver program interventions in the most effective means possible, by leveraging state-of-the-art technology with the goal of a highly engaging user experience. Content includes self-management educational curriculum, defined as a coordinated set of educational experiences with specific learning outcomes and employing teaching strategies that are dynamic and reflect current evidence and best practice guidelines [20]. Message content is derived from the educational curriculum, may target the intended behavior, and appeal to the user. Messages may address key benefits and potential obstacles as well [36].

##### Derivation

Features, functionality, and content incorporated into the app were informed by the aforementioned domains of the framework and developed using an iterative design process. Features and functionality evolved with technology advances, but were always guided by the objectives and intent of the defined set of interventions. New innovations were also considered if it appeared that they could facilitate intervention objectives. For example, a GPS-facilitated feature to identify nearby restaurants and their menus to support meal planning and healthy eating was added to the diabetes app. Table 1 provides examples of the intervention plan that informed the design of product features for the diabetes app.

**Table 1:** Diabetes mHealth interventions mapped to features.

Intervention type	Intervention description	Product features/content
Educational and skills-building support	Educational and skills-building curriculum content that can be delivered universally to all issues or customized based on individual user data.	Universal education videos, tips
Monitoring support	Guidance for structured blood glucose monitoring with regards to activity types, timing, and frequency of data collection and visual displays of data collected	Logbook (journal blood glucose values, carbs, physical activity); structured blood glucose checking feature
Coaching support	Tailored real-time feedback and trending messages based on customized care-plan prioritized behaviors.	Real-time feedback; longitudinal feedback; customized delivery of video content
Behavioral adherence support	Behavioral adherence tool-set pushed to support customized-care plan. Adherence tools may be accompanied by coaching to address associated self-identified barriers to and motivators for action.	Medication adherence tools (medication list, medication schedule, medication reminders); carb estimation tool; restaurant locator
Patient-provider communication support	Reports sent to health care team with tailored content based on system analysis (eg, patient data inputs and specific provider recommendations) to facilitate patient-provider discussions.	Tailored health care provider report
Patient engagement	Product features, functionality, and content developed specifically to encourage product use.	Homepage design; time-based “touchpoint” messages

### Application and Refinement of the Framework

The framework was applied to the development of integrated clinical/behavioral interventions for use in the development of mobile apps for 7 chronic diseases (diabetes, epilepsy, asthma, chronic obstructive pulmonary disease, lupus, HER2+ breast cancer, and low back pain). The findings from this process served as the basis for a chronic disease mHealth intervention taxonomy for scalable design. Table 2 lists the details of the taxonomy. The attributes of the domains were informed by market research with various groups (health care providers, patients, SMEs, caregivers, large pharma) and evidence-based literature (national clinical guidelines, peer-reviewed articles, best practices) that were determined to be valuable in the management of chronic disease.

**Table 2:** Chronic disease mHealth app intervention design taxonomy.

Domains	Attributes
Value drivers	Clinical; behavioral; psychosocial (quality of life); health care costs; patient engagement in their healthcare
Outcomes and metrics	Clinical; behavioral; quality of life; intermediate outcomes (eg, knowledge); patient engagement
Key stakeholders and program objectives	Patient; health care team; caregiver; social support community; improve diabetes self-management; improve clinical decision-making; improve patient-provider communication
Essential behaviors	Medication-taking; monitoring; problem solving; eating; reducing risk (complications); being active; healthy coping; patient engagement; patient-provider communication
Multidimensional profile	Clinical; behavioral; psychosocial
Clinical/behavioral interventions	Education and skills-building support; monitoring support; coaching; behavioral adherence support; time management support; problem-solving support; patient-provider communication support; social support
Features/functionality/content	Logbook/journal/tracker; self-management tools; reminders; alerts; calendar/scheduling features; patient-provider discussion guide; patient data summary reports; online social network; interactive tutorials/guidance *Educational content:* standard (guideline-based content); customized *Message content:* prompt time-based; real-time feedback; trending feedback

In total, 5 principal programmatic value drivers and 3 program objectives for key stakeholders emerged. There were variations in patient self-management objectives, ranging from single aspects of self-management to comprehensive self-management programs.

Informed by the American Association of Diabetes Educators behavioral domains (AADE7), a total of 7 health behavioral domains were validated across diseases. However, specific essential behaviors facilitated by the mHealth product within each of the domains were specific to the disease (eg, self-monitoring of blood glucose versus self-monitoring of seizure activity). Two additional patient behavioral domains emerged from the process: (1) a health behavioral domain (engaging in health care visits/communicating with the health care team), and (2) initial and ongoing engagement in mHealth product use, which emerged as critical from a program implementation perspective. An analysis of the multidimensional profile for appropriate segmentation of delivered interventions and content yielded 3 principal profile dimensions to be addressed during the first stages of program implementation.

Overall, 8 core evidence-based intervention types identified in the literature were validated via the iterative product development cycle. Additional interventions were incorporated into strategies based on formative and Voice of the Customer marketing research. For example, for the epilepsy app, participants were specific about avoiding the term “epileptic”; for the HER+2 cancer app, participants only wanted information specific to their type of cancer and not breast cancer in general. Also, any participants linking to a social community wanted the participants of that community to have the same specific disease (eg, type 2 versus type 1 diabetes). Patients indicated the importance of these interventions for ongoing engagement with mHealth products. Interventions could include messages that were based on clinical guidelines and/or simply designed to enhance engagement, depending on condition and program objectives. For example, in the diabetes app, real-time feedback messaging served to provide clinically relevant content for the user, and in the epilepsy app, it was used to engage the user in tracking information.

### Using the Finalized Framework for Product Development

The “waterfall” framework was applied to the design of an FDA-cleared diabetes prescription app for type 2 diabetes self-management. Table 3 links the design elements to the framework domains.

**Table 3:** Application of the framework to the mHealth prescription product (stakeholder input on the Diabetes mHealth App)^a,b^.

Framework domain		Design elements
Value drivers		Clinical behavioral engagement
**Outcomes and metrics**		
	Clinical^c^	*Outcome:* At target or improved *Metrics:* A1c<7% or 8% (risk) or 1% improvement if above; BP<140/90 or decrease 10/5 mm/Hg if above; LDL<100 mg/dL or <70 (cardiac risk) or a decrease of 26% if above; HDL>50 mg/dL for females or an increase of 8% if above; HDL>40 mg/dL for males or an increase of 8% if above; Triglycerides<150 mg/dL or a decrease of 26% if above
	Behavioral^d^	*Outcome:* Improve or maintain medication adherence *Metric:* Medication adherence: ≥80% adherence to metabolic meds (correct meds and as prescribed)
	Patient Engagement^e^	*Outcome:* Initial mHealth application use*.* Sustained mHealth application use *Metrics:* One interaction with the application each week for 1^st^ 3 months
Program objectives for stakeholders		Effective diabetes self-management; Effective clinical decision-making; Effective patient-provider communication
Essential behavioral domains/supporting actions		AADE7^f^; medication-taking; monitoring (BG^g^); problem-solving (high & low BGs); eating; reducing risk (complications); being active; healthy coping; engagement
**Multidimensional profile**		
	Clinical	Medication regimen; BG value ranges
	Contextual	BG reading type
	Behavioral	Medication-taking behaviors: consistent, inconsistent, nonadherent
EBG-driven clinical and behavioral interventions (evidence-based)		Educational and skills-building support; self-monitoring of blood glucose support; coaching support; behavioral adherence support (medication-taking, carb counting); patient-provider communication support; problem-solving support (eg, addressing high and low BGs); social support
**App features/content**		
		Logbook; medication list & schedule; carb estimation tool; SMBG^h^ tool; reminders; alerts; patient data summary report for health care team; clinical decision support feature
	Information/educational content	Learning library; ADA^i^ standards of care; DSME/S^j^ educational topics; content to support AADE7 self-management behaviors; tips
	Message types	*Reminders Time-based “touch point messages”:* daily messages for engagement, motivation *Longitudinal feedback messages:* based on multiple BG data points entered into journal to improve skills for SMBG *Real-time feedback:* Based on data entered into journal to improve skills for SMBG

^a^ Implementing organization = health care technology company

^b^ Program implementation model = Rx only by health care providers for patients

^c^ Clinical outcomes for chronic conditions (at the patient level)

^d^ Behavioral outcomes related to patient self-management

^e^ Initial and sustained mHealth application use

^f^ AADE7: American Association of Diabetes Educators behavioral domains

^g^ BG: blood glucose

^h^ SMBG: self-monitoring of blood glucose

^i^ ADA: American Diabetes Association

^j^ DSME/S: Diabetes self-management education and support

## Discussion

The framework resulted in a systematic, replicable, and scalable mechanism for designing mHealth product features, functionality, and content to improve health outcomes in real-world settings for a range of chronic diseases. Through iterative research, design, and testing processes, the “waterfall” framework was created to provide a mechanism for translating evidence and research across multiple disciplines. This framework can be used to drive the systematic development of apps designed to provide different levels of intervention support, from tracking to comprehensive care management for various diseases.

In order for mobile technology to achieve its promise of revolutionizing health care, we must determine which aspects of mHealth work, for which diseases, for whom, and to achieve which outcomes. This framework can provide a standard approach to design and evaluate the effectiveness of health care apps and to inform policy, practice, and research.

From a health care policy and regulation perspective, if mobile technology is to be used as a form of therapy, it is critical to have a framework that translates existing guidelines and practices into mHealth products in a transparent, universal, and standardized way. Such a framework creates a mechanism that connects guidelines, health care practices and programs, user interface and experience, and mobile technology capabilities for the design of mHealth products.

The development of the framework also shifted the paradigm from feature-based to programmatically driven mHealth design, providing interdisciplinary product teams with a common understanding of the goals and objectives of product features and functionality. The framework influences decisions related to UI/UX, and how to ensure a user experience that integrates support into a person’s daily self-care activities. Finally, the framework has the potential to adapt to evolving health care service delivery systems, informing the design of interventions that drive outcomes which support new initiatives.

The framework offers an avenue for researchers to understand how and where guidelines, standards of care, and evidence can be advantageous for mHealth design. It facilitates iterative design and testing during the concept development phase, and process and impact evaluation, which can be used to inform future design. Future applications should validate the framework in diverse service delivery settings so that it can be refined based on broader utilization.

Mobile health technology creates a shift in the paradigm of chronic disease management. It offers new possibilities to engage patients in self-management of their chronic diseases in ways that did not exist in the past. To maximize the potential of mHealth requires the integration of research and expertise from multiple disciplines including clinical, behavioral, data analytics, and technology to achieve patient engagement and health outcomes. This paradigm shift also triggers a need for new approaches to designing clinical and behavioral support for chronic disease management that can be implemented through existing health care services and programs.

The Chronic Disease mHealth App Intervention Design Framework domains and the corresponding design elements developed through this process may provide a foundation for the digital health industry to systematically expand mobile health interventions and validate their effectiveness across multiple implementation settings and chronic diseases. Further enhancement and validation of the framework is needed to recognize these benefits.

## Abbreviations

AADE7: American Association of Diabetes Educators seven behavioral domains
BG: blood glucose
FDA: Federal Drug Administration
mHealth: mobile health
SME: subject matter experts
UI/UX: user interface/user experience
